# Public health genomics research in Italy: an overview of ongoing projects

**DOI:** 10.3389/fpubh.2024.1343509

**Published:** 2024-02-21

**Authors:** Erica Pitini, Valentina Baccolini, Claudia Isonne, Paola Maran, Carolina Marzuillo, Paolo Villari, Daniela Galeone, Francesco Vaia

**Affiliations:** ^1^Directorate-General for Health Prevention, Ministry of Health, Rome, Italy; ^2^Department of Public Health and Infectious Diseases, Sapienza University of Rome, Rome, Italy; ^3^Department of Translational and Precision Medicine, Sapienza University of Rome, Rome, Italy

**Keywords:** public health genomics, research, precision medicine, precision public health, governance, Italy

## Abstract

Public health genomics (PHG) aims to integrate advances in genomic sciences into healthcare for the benefit of the general population. As in many countries, there are various research initiatives in this field in Italy, but a clear picture of the national research portfolio has never been sketched. Thus, we aimed to provide an overview of current PHG research projects at the national or international level by consultation with Italian institutional and academic experts. We included 68 PHG projects: the majority were international projects in which Italian researchers participated (*n* = 43), mainly funded by the European Commission, while the remainder were national initiatives (*N* = 25), mainly funded by central government. Funding varied considerably, from € 50,000 to € 80,803,177. Three main research themes were identified: governance (*N* = 20); precision medicine (PM; *N* = 46); and precision public health (*N* = 2). We found that research activities are preferentially aimed at the clinical application of PM, while other efforts deal with the governance of the complex translation of genomic innovation into clinical and public health practice. To align such activities with national and international priorities, the development of an updated research agenda for PHG is needed.

## 1 Introduction

Public health genomics (PHG) is an attempt to responsibly and effectively translate the rapid increases in genome-based knowledge and technologies into population health benefits (1). Recent decreases in sequencing costs and the marked progress in data science present many opportunities for the incorporation of genomic information into new strategies for clinical and public health practice. Precision medicine (PM) focuses on identifying the most effective medical intervention for patients based on their genetic and biochemical profile, as well as specific environmental and lifestyle factors (2). Impressive advances have been made in cancer, where next generation sequencing has enabled the prediction of cancer susceptibility, sensitivity to therapy, prognosis and residual disease (3). Moreover, the use of population level data on genomics and other health determinants is expected to improve health outcomes at the population level, paving the way for a field called precision public health (PPH) (4). Some emerging applications of PPH are the development of more intensive screening programs for people at greater risk of cancer, and the detection and investigation of infectious disease outbreaks (5).

To realize the full potential of these promising approaches, many countries are fostering research initiatives aimed at driving the development of public policies, guidelines and health programs for the implementation of evidence-based genomic applications (6–8). Italy is no exception, although research activities seem to have begun in a disorganized way. The task of assuring coordination of national efforts in PHG has recently been entrusted to the Inter-institutional Committee for PHG (IC), a national board established in 2022, which includes representatives from the Italian Ministry of Health (MoH), the Italian Institute of Health (ISS), the National Agency for Regional Healthcare Services (AGENAS), the Italian Medicine Agency (AIFA), and the Italian Regions, in addition to academic experts in the field (9).

In particular, the IC is responsible for supervising PHG activities throughout the country and evaluating their alignment with European priorities. For its first assignment, the IC aimed to investigate the current state of development of PHG research in Italy. Since no comprehensive repository for national PHG projects is available, a mapping exercise was performed. The aim of this paper is to describe the methods and the results of an overview of the current PHG research portfolio in Italy and to discuss its main policy implications.

## 2 Methods

### 2.1 Subject of investigation and inclusion criteria

This overview included any research project involving PHG that started or was ongoing in Italy during 2022 (January 1st–December 31st). Both national projects and international projects in which Italian researchers participated were eligible for inclusion, whereas projects sponsored only at a regional level (i.e., involving one or more Italian Regions without a national commitment) were excluded. For the purposes of this study, PHG was defined as “a multidisciplinary field concerned with the responsible and effective translation of genomic science and technologies into clinical and public health practice” (10).

### 2.2 Data collection

We retrieved candidate projects by email consultation with the Italian IC, which comprised 39 expert representatives from the following institutions: I. Italian MoH (*N* = 26, from seven different Directorates-General); II. ISS (*N* = 2); III. AGENAS (*N* = 2); IV. AIFA (*N* = 2); V. Italian Regions (*N* = 4); VI. Italian universities (*N* = 3, leaders in the field of PHG from three different universities).

The experts were provided with the details of the overview during an online meeting and were asked to participate in the design of the project tracker tool (PTT). A draft of the PTT was e-mailed to the experts and their feedback was used to improve it. The final PTT consisted of an Excel data sheet collecting the following information for each project: title; start/end date; objectives; funder; lead institution; number of implementing partners; number of countries involved; funding; referring website; involvement of the IC (Supplementary Table S1).

In May 2022, we officially started data collection by emailing the experts with the PTT and the instructions for completion. To ensure the completeness of the results, we asked the experts to continuously update the PTT with newly funded research projects as they became available over the year. Data collection was closed at the end of December 2022. Reminders were issued during IC meetings and were also sent twice by e-mail during the year.

### 2.3 Project selection and data synthesis

For each project selected, unclear or missing information was clarified or retrieved by exploring official websites (Supplementary Table S2). Two researchers removed duplicates and screened the projects according to the inclusion criteria. Projects that clearly did not meet the eligibility criteria were excluded.

A descriptive analysis of the projects included was performed, using frequencies, percentages and ranges. Moreover, according to their primary aim, projects were mapped to three thematic categories and six sub-categories, as follows: (I) governance, further divided into (Ia) networking and coordination for innovation, (Ib) data and infrastructure, (Ic) adoption of health technology; (II) PM, further divided into (IIa) cancer and (IIb) non-oncological diseases; and (III) PPH, including only one sub-category, namely (IIIa) surveillance of infectious diseases. Topic attribution was cross-checked by three reviewers.

## 3 Results

### 3.1 Project selection

**Figure 1:**
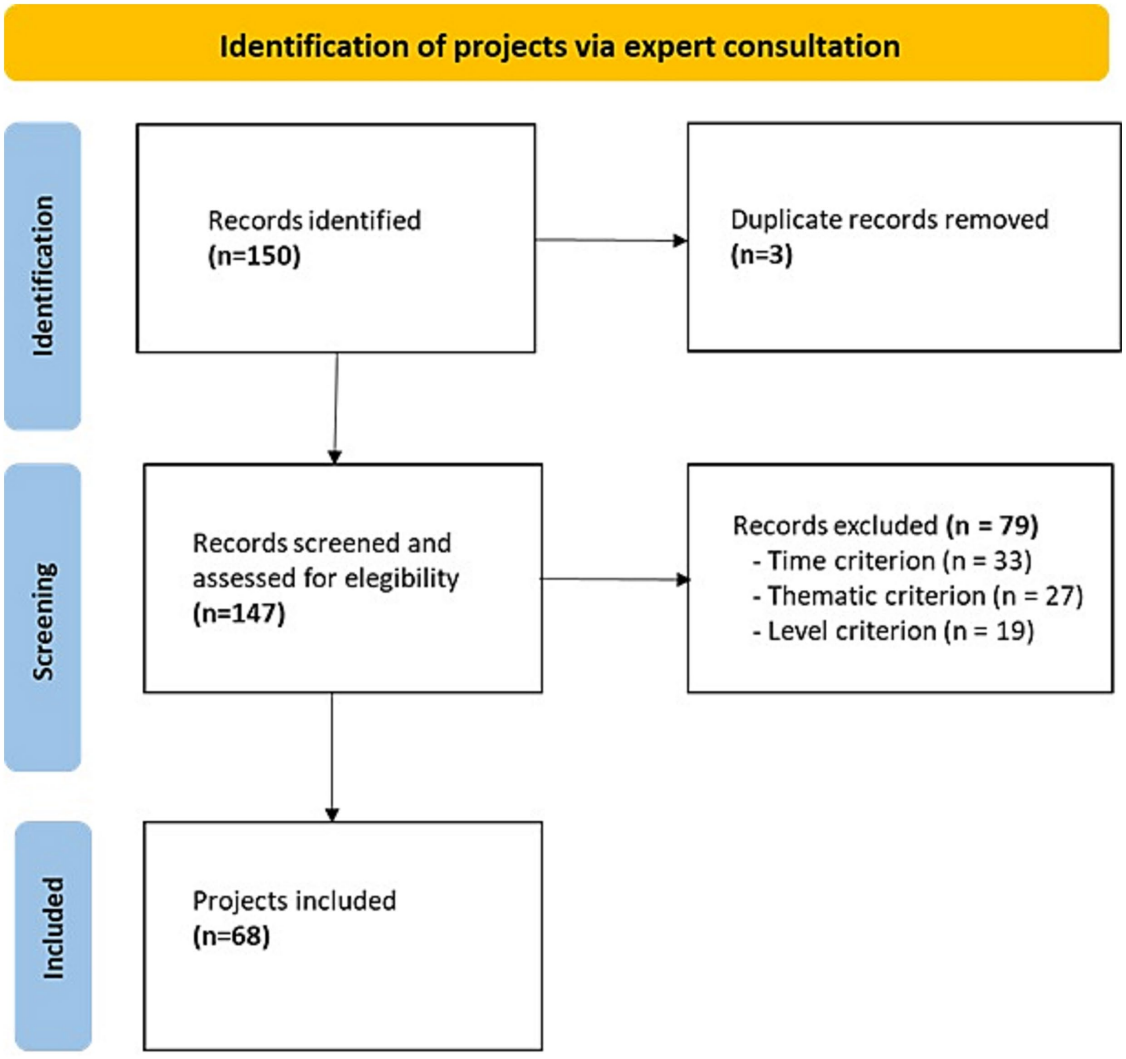
PRISMA flow diagram.

After removal of duplicates, a total of 147 projects resulted from the consultation with IC members (Figure 1). Screening by inclusion criteria selected 68 projects (11–70) (Supplementary Table S2). The reasons for exclusion were: projects not in progress during the year 2022 (*N* = 33; concluded before 2022 or yet to start); projects off-topic (*N* = 20; mainly basic research) or topic not clear due to lack of information (*N* = 7); and projects sponsored only at the regional level (*N* = 19).

### 3.2 Project characteristics

#### 3.2.1 General features

The characteristics of the 68 projects included in the study are extremely variable. Thus, with regard to timing, they were launched between the years 2017 and 2022, and are expected to conclude between 2022 and 2029 (Supplementary Table S2), with a duration of 1 to 10 years (median: 3, interquartile range: 3–4.5). The number of countries involved ranges from 1 (Italy alone) to 20 (median: 4, interquartile range: 1–6), while the number of partners involved ranges from 1 (one Italian participant) to 84 (large multi-country projects; median: 6, interquartile range: 4–15). The total amount of funding ranged from € 50,000 to € 80,803,177 (median: € 871,820 interquartile range: € 471,000 – € 4,000,000).

#### 3.2.2 Funders and coordinators

There are markedly more international (*N* = 43) than national (*N* = 25) projects (Table 1; Supplementary Table S2). with almost all the international ones being funded by the European Commission (EC; *N* = 40), while the main funder of national projects is central government (*N* = 14). A small number of national projects are funded by bank groups (*N* = 2), while the remaining national and international projects are financed by non-profit organizations (*N* = 12).

**Table 1:** Description of the projects retrieved (*N* = 68).

Project characteristics	*N*	Percentage
*Level*		
National	25	36.8
International	43	63.2
*Funding organization*		
European Commission	40	58.8
National central government	14	20.6
Non-profit organization	12	17.6
Bank group	2	2.9
*Coordinator*		
Healthcare facility	26	38.2
University	23	33.8
Governmental research organization	11	16.2
Non-profit research organization	7	10.3
For-profit research organization	1	1.5
*Theme*		
Governance	20	29.4
Networking and coordination for innovation	9	13.2
Data and infrastructure	9	13.2
Health technology adoption	2	2.9
Precision medicine	46	67.6
Cancer	29	42.6
Non-oncological disease	17	25.0
Precision public health	2	2.9
Surveillance of infectious diseases	2	2.9

N, number.

Overall, the retrieved projects are mostly coordinated by healthcare facilities (*N* = 26) and universities (*N* = 23; Table 1; Supplementary Table S2), with the remainder being managed by research organizations, either governmental (*N* = 11) or non-governmental (*N* = 8), most of the latter being non-profit (N = 7). Of the international projects, one quarter are coordinated by an Italian institution (*N* = 17). Finally, almost all of the retrieved projects count among its funders or executors at least one of the Institutions represented in the IC (*N* = 63).

#### 3.2.3 Theme

The projects were mapped to three thematic categories, namely (I) governance (*N* = 20); (II) PM (*N* = 46); and (III) PPH (*N* = 2; Table 1; Supplementary Table S2).

(I) Governance

The 20 projects in the governance category aim to ensure a coordinated approach to the long-term implementation of genomics for the personalization of healthcare (Supplementary Table S2). They can be further classified into three sub-categories (Table 1).

(Ia) Networking and coordination for innovation

The first sub-category, “Networking and coordination for innovation,” includes nine EU-funded projects aimed at fostering collaboration across European countries and beyond, with special regard to the development of training programs for researchers and healthcare professionals, the identification of recommendations for the harmonization of research and implementation initiatives, and the engagement of relevant stakeholders (including citizens, patients, healthcare professionals, policy makers and private companies; Supplementary Table S2). In Italy, an important leading role is played by the Catholic University of the Sacred Heart of Rome, which coordinates three of these projects funded through EU Horizon programs. The first is the ExACT (European network staff eXchange for integrAting precision health in the health Care SysTems) project, which involves eight member states (MS) plus the United Kingdom (UK), Canada and the United States, working together since 2019 to train a new generation of precision health professionals through a five-year secondment plan (16). The second is PROPHET (A PeRsOnalized Prevention roadmap for the future HEalThcare), a four-year project involving 12 EU MS plus the UK, launched in 2022 to develop a strategic roadmap for the implementation of innovative, sustainable and effective personalized programs to prevent common chronic diseases (27). Finally, the four-year IC2PerMed (Integrating China in the International Consortium for Personalized Medicine) project was launched in 2020 with the specific aim of fostering EU-China cooperation in the field of personalized medicine. This project is managed by the International Consortium for Personalized Medicine, a MS-driven initiative of over 40 international ministries and funding agencies (20).

(Ib) Data and infrastructure

The second sub-category, “Data and infrastructure,” includes nine projects aimed at developing infrastructure, tools and regulatory frameworks for the collection and use of genomic data (Supplementary Table S2). The key driver of this sub-category is the 2018 One Million Genomes Initiative (1 + MG), which is committed to creating a European data infrastructure for genomic and clinical data to support research, personalized healthcare and health policy formation (71). In fact, Italy is involved in both EU-funded projects launched to realize 1 + MG initiatives, coordinated by ELIXIR, a European intergovernmental organization. The first is the three-year B1MG (Beyond 1 Million Genomes), launched in 2020 to provide coordination and support to the 1 + MG initiative by defining the infrastructure, data standards and legal guidance for cross-border access to genomic data (11). The second is the 2022 GDI (Genomic Data Infrastructure) project, which will implement the recommendations of B1MG to create and deploy the technical capacity for accessing genomic data by 2027 (17). Since the end of 2021, Italy has further supported the fulfillment of these European goals through a dedicated two-year national project financed by the MoH-National Centre for Disease Prevention and Control and coordinated by Sapienza University of Rome, entitled “Italian Genomic Strategy” (30). Moreover, the ten-year project “Health Big Data,” dedicated to the deployment of an IT platform for sharing genomic and clinical data between the national Scientific Institutes for Research, Hospitalization and Healthcare, has been funded by the Italian Ministry of Economy and Finance since 2019 and is coordinated by a national oncology network named Alliance Against Cancer (19).

(Ic) Health technology adoption

Finally, the third sub-category, “Health technology adoption,” embraces two projects, one Italian and one European, that focus on guiding genomic-technology acquisition and use through health technology assessment (HTA) and procurement, respectively (Supplementary Table S2). The Italian project was financed at the end of 2019 by the MoH for a period of two years to design a comprehensive national path for the HTA of genetic and genomic tests, including the three phases of priority setting, assessment and appraisal (14); the European OncNGS (NGS diagnostics in 21st century oncology: the best, for all, at all times) project, launched in 2020 with the participation of eight buyers from five EU MS, coordinated by researchers in Belgium, will prepare a pre-commercial procurement procedure to provide the best next-generation sequencing diagnostic technologies for all solid-tumor and lymphoma patients by 2026 (25).

(II) Precision medicine

PM is the largest category and comprises 46 projects aimed at assessing the use of genomic information to provide a more precise approach to diagnosis, prognosis and treatment of disease (Supplementary Table S2). In particular, the majority of these projects aim to identify molecular biomarkers that predict the course of a disease and the response to treatment. While customizing care using a combination of clinical and genomic factors is not unusual among the projects in this study, very few also consider lifestyle and environmental factors. PM projects can be further classified according to the disease of interest (Table 1).

(IIa) Cancer

**Figure 2:**
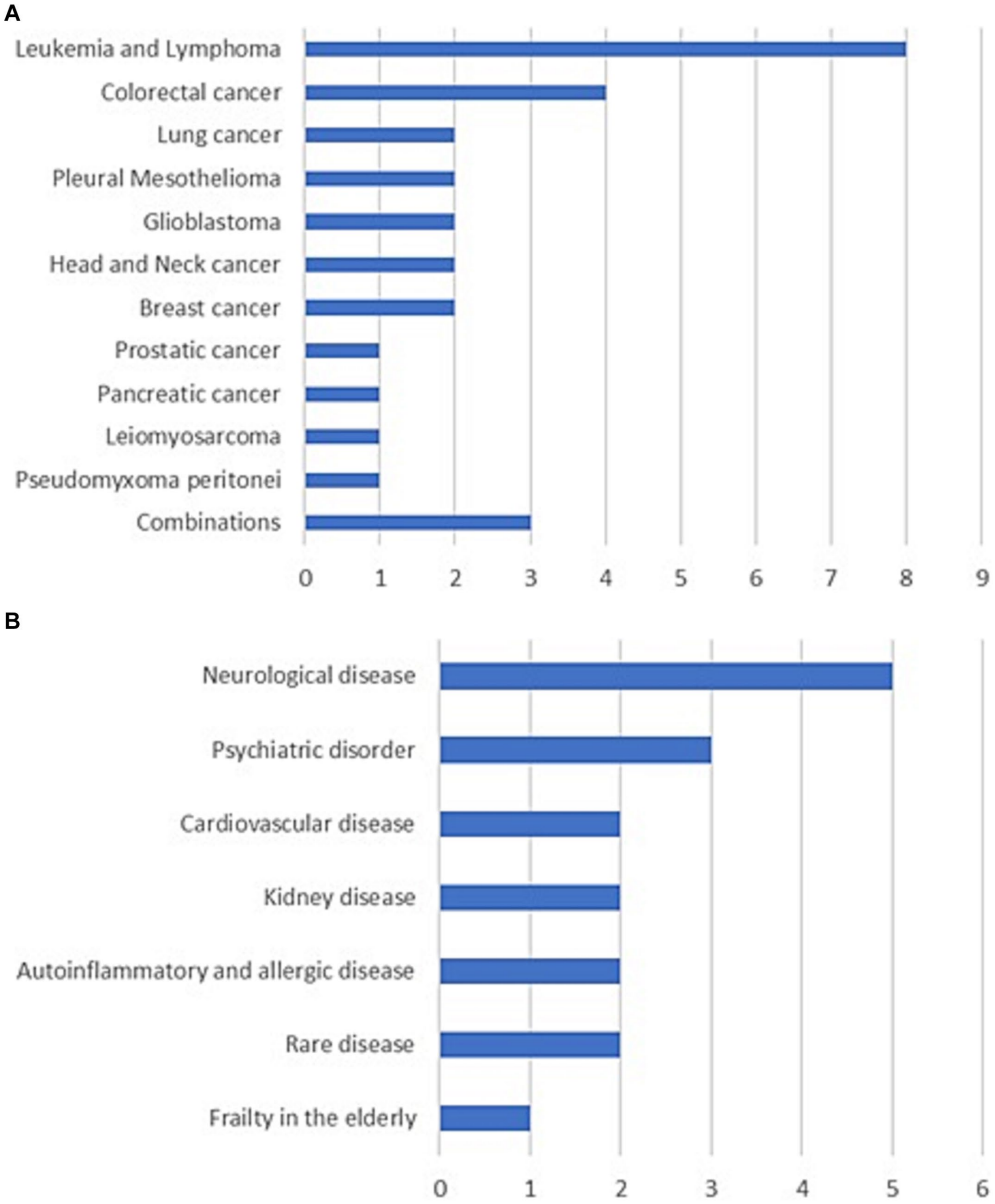
Number of precision medicine projects by disease. **(A)** Cancer. **(B)** Non-oncological diseases.

Over half of the PM projects focus on cancer (*N* = 29), especially hematological cancers and gastrointestinal cancers (Figure 2A; Supplementary Table S2). Among the international projects coordinated by Italy, one example is the three-year project IMAGene (Epigenomic and machine learning models to predict pancreatic cancer), supported by ERA PerMed, a funding scheme for personalized medicine research projects cofounded by the EC (49). IMAGene, which involves participants from six MS and is coordinated by the European Institute of Oncology in Milan, commenced in 2022 to develop, implement and test a comprehensive Cancer Risk Prediction Algorithm for the early detection of pancreatic cancer in high-risk asymptomatic subjects, based on germline mutations, DNA methylation profiling and magnetic resonance imaging. Among the national projects, one that has attracted many participants is the 2019 GerSom (Germline Somatic Panel) project, which is funded by the MoH for a period of three years under the supervision of Alliance Against Cancer (47). It aims to improve the management of patients with ovarian, breast and colorectal cancer through the validation of a genomic panel for somatic and germline variants, which allows the diagnosis of both genetic risk and sensitivity to new drugs.

(IIb) Non-oncological diseases

Of the projects on non-oncological diseases (*N* = 17), the largest group addresses neurological diseases (*N* = 5), especially multiple sclerosis (Figure 2B; Supplementary Table S2). Among the international projects coordinated by Italy, there is another three-year ERA PerMed project named FindingMS (An integrated approach to predict disease activity in the early phases of multiple sclerosis), launched in 2019 with the participation of three MS and coordinated by the San Raffaele Research Hospital of Milan (45). It aims to design a predictive algorithm of disease activity for multiple sclerosis based on genetic and environmental factors, which will enable personalized treatment from the early phases of the disease. At the national level, a notable example is the 2022 NEUDIG (Unveiling the hidden side of NEUrodevelopmental DIsorder Genetics) project, funded by the Ministry of University and Research for a period of three years and coordinated by the University of Genova (38). Its purpose is to strengthen the molecular diagnosis of neurodevelopmental disorders by non-conventional genomic, transcriptomic, and functional analyses.

(III) Precision public health

Only two projects mapped to the PPH category, i.e., the use of genomic technologies to improve public health policy and practice (Supplementary Table S2). They are two national projects, funded by the Italian MoH-National Centre for Disease Prevention and Control in 2020 and 2022 for a period of two years each. They both focus on the same thematic sub-category, i.e., the molecular surveillance of infectious viral diseases (Table 1): while the first project, “Molecular characterization of SARS-CoV-2 in Italy,” coordinated by the ISS, is aimed at monitoring the SARS-CoV-2 virus in the country, by both time and geographical area, through genomic analysis (70), the other, i.e., SURVEID (SURVeillance of Emerging Infectious Diseases), coordinated by the Experimental Zooprophylactic Institute of Lombardia and Emilia-Romagna, plans to test a metagenomics next generation sequencing diagnostic platform for the surveillance of emerging viral disease threats (30).

## 4 Discussion

This paper highlights the main features of current PHG research in Italy by the collection and analysis of national and international research projects on the topic. Overall, PHG research in Italy seems to be mainly funded by the EC and is managed by healthcare facilities and universities. Notably, three main research themes for the integration of genomics into healthcare were identified: governance, PM and PPH. The predominant theme appears to be PM, mainly in its narrowest sense, as most PM projects aim to explore the use of molecular biomarkers to guide clinical decision making, with a particular focus on therapeutic choices for cancer patients (72). By contrast, only a few PM projects embrace a broader approach, as they combine the assessment of genomic and environmental or lifestyle determinants to guide the management of patients, more often in the field of non-oncological diseases (73). While PM, in its various facets, dominates the current research portfolio, PPH is clearly the least active research area. According to our results, in Italy the integration of genomics into public health strategies has probably been boosted by the Covid-19 pandemic, as the two PPH projects retrieved relate to the genomic surveillance of viral infectious diseases, including Covid-19. In the meantime, considerable efforts seem to be underway to guarantee the governance and sustainability of the long-term implementation of PM, the third key research theme emerging from the overview. In particular, major investments in governance are being made to foster partnership and collaboration across European countries to tackle two main implementation challenges: (i) ensuring the alignment of research activities and workforce education; and (ii) enabling the sharing and use of large-scale genomic data.

To the best of our knowledge, this overview is the first attempt to portray the Italian PHG research portfolio over a specific period of time, with the ultimate aim of informing relevant national decision makers. Nevertheless, it has to be considered that, although most of the projects identified will take months and even years to conclude, the growing number of PHG research projects funded each year may rapidly change the scenario we outline here. Thus, it would be useful to regularly update the database to monitor any potential change in research investment. To this end, designing a national or international repository for PHG research projects would surely be appropriate, as this would reduce duplicated effort, improve transparency, highlight opportunities for funding and monitor progress in this quickly evolving area.

Moreover, it would be interesting to understand whether the currently funded research activities align with national and international reference standards in the field. Unfortunately, we are not aware of any updated benchmark recommendations for PHG research, either nationally or at the European level. Although the “National plan for the innovation of the Health System based on omics sciences,” published in 2017, identified seven research opportunities for the integration of omic sciences into the National Health Service, these are likely to be outdated. The opportunities identified in 2017 were: (I) Big data and computational medicine; (II) Health literacy of citizens and healthcare professionals; (III) Drug repositioning and pharmacogenomics; (IV) Primary prevention of chronic disease; (V) Secondary prevention of breast cancer; (VI) Early detection of cancer; and (VII) Undiagnosed rare diseases (74). More recently, given the need to update the plan, the National Health Council issued its own recommendations for new priorities to be addressed (75). With regard to genomics research, the main indication is to further investigate the complex interactions between genetic and non-genetic factors in the pathogenesis of disease and to include non-genetic factors in risk-assessment algorithms. This resolution is consistent with our results, as the role of environmental and lifestyle factors appears to be neglected in ongoing Italian research.

At the European level, we found no official documents on shared research priorities for PHG. Some very general directions are provided by a policy briefing recently published in the context of the 1MG project (76). The first aim of the document is to set out policy recommendations for the implementation of genomics in healthcare, some of which also concern the field of research. It is recommended that close cooperation between clinical, research and industrial partners be established to ensure that the latest advances in science and technology are captured as they arise, and that research and clinical outcomes are coordinated. A further recommendation is to implement a data management plan to facilitate sharing of genomic and health information for clinical and research purposes at regional, national and international levels. According to our results, these two proposals are already being pursued in the research projects currently ongoing in Italy.

The main limitation of our work is in the comprehensiveness of the overview, which is affected by two conditions. First, we included only projects reported by members of the IC. Indeed, nearly all projects benefit from the involvement of at least one of the consulted institutions, as a funder or executor. However, since the IC includes the main national governmental and academic institutions involved in PHG, it is expected to account for most of the ongoing activities. Nevertheless, it is worth mentioning that a government funding organization not included in the IC emerged from the overview, namely the Ministry of Research (MoR). Indeed, the commitment of the MoR to PM is being strengthened by its recent decision to assign part of the research funds provided by the National Recovery and Resilience Plan to a nationwide research partnership, called HEAL ITALIA, which aims to create a Health Extended ALliance for Innovative Therapies, Advanced Lab-research, and Integrated Approaches of Precision Medicine (77). Thus, although it is not purely a healthcare institution, the future involvement of the MoR in the IC could be considered. Second, as stated in the Methods, we included only projects financed on either an international or a national scale. In fact, the Italian national healthcare system is decentralized to 21 regional healthcare systems, with different degrees of autonomy, and just four Regions participate in the IC on behalf of all the others, making the search for regional projects flawed by a potential selection bias. For this reason, we tried to avoid the tangle of regionally funded projects by focusing on centrally funded efforts in PHG research.

As for the accuracy of the evidence provided, we confirmed all data by an internet search of official websites. Nevertheless, we cannot exclude the possibility that in some cases a lack of clear information on the projects’ aims, together with their cross-cutting nature, may have affected their inclusion or assignment to particular thematic categories, even though both the project selection and data collection were performed by at least two authors. Lastly, it should be noted that the present overview made no attempt to assess the quality of the projects retrieved or whether they met their objectives, as this was not our goal.

In conclusion, we have provided an overview of the national research portfolio in PHG in Italy, with the primary aim of informing policy makers and fostering the coordination of national efforts for the implementation of evidence-based genomic applications. We found that research investments, mainly supported by the EC, are preferentially aimed at the clinical application of PM, but significant endeavors are also underway on the governance of the complex translation of genomic innovation into clinical and public health practice. Nevertheless, this is only the first step on a challenging path toward a coordinated and sustainable research agenda. First of all, this overview should be regularly updated to keep up with the systematic launch of new research projects. Then, updated research plans should be developed to align national activities with national and international priorities and avoid unaddressed needs and waste of resources.

## Data availability statement

The original contributions presented in the study are included in the article/Supplementary material, further inquiries can be directed to the corresponding author.

## Author contributions

EP: Writing – original draft, Conceptualization, Data curation. VB: Writing – review & editing Data curation. CI: Data curation, Writing – review & editing. PM: Investigation, Writing – review & editing. CM: Project administration, Writing – review & editing. PV: Supervision, Writing – review & editing. DG: Conceptualization, Writing – review & editing. FV: Supervision, Writing – review & editing.
