# Supplementary material for: Public health genomics research in Italy: an overview of ongoing projects
Source: Front Public Health. 2024 Feb 21;12:1343509. doi: 10.3389/fpubh.2024.1343509 (PMC10915058; doi:10.3389/fpubh.2024.1343509)
Supplement: Supplementary file 1 [file Table_1.DOCX]

Supplementary Material

# Supplementary Table

Table S1 – Definition of the data fields of the Project Tracking Tool

| Data field | Definition |
| --- | --- |
| Title | Title of the research project |
| Start date | Date the project started |
| End date | Date the project will end |
| Objectives | General and specific objectives of the project |
| Funder | Name of the main organisation funding the project |
| Lead institution | Name of the organisation leading the project |
| N. of implementing partners | Number of organizations participating in the project |
| N. of involved Countries | Number of countries participating in the project |
| Amount of funding (€) | Total amount awarded to the project (including co-funding) |
| Referring website | Official website of the project |
| Involvement of the IC | Whether at least one IC body is involved in the project at any level |

N: number; IC: Inter-institutional Committee for Public Health Genomics

Table S2 – Project list and main features (alphabetical order)

| Title | Level | Funder | Start year | End year | Coordinator | Theme ^1^ | Web-site |
| --- | --- | --- | --- | --- | --- | --- | --- |
| 3TR: Identification of the Molecular Mechanisms of non-response to Treatments, Relapses and Remission in Autoimmune, Inflammatory, and Allergic Conditions | International | European Commission | 2019 | 2026 | Governmental research organization | IIb | <https://cordis.europa.eu/project/id/831434> |
| 3Tx3N: tre team per migliorare diagnosi e cura dei tumori al seno TRIPLI-Negativi | National | Bank group | 2021 | 2023 | Non-profit research organization | IIa | <https://www.fondazionetempia.org/risultati-promettenti-dallo-studio-sui-tumori-al-seno-tripli-negativi/> |
| A Functional Precision Medicine Platform in Adult Leukemia | National | Non-profit organization | 2022 | 2023 | University | IIa | <https://www.ifabfoundation.org/ifab-activities/projects/a-functional-precision-medicine-platform-in-adult-leukemia> |
| A genomic-driven diagnosis to deliver bespoke therapeutic strategies in HER2-low breast cancer patients | National | Non-profit organization | 2020 | 2024 | Healthcare facility | IIa | <https://www.airc.it/ricercatori/i-nostri-ricercatori/caterina-marchi%C3%B2> |
| ACRCelerate: Colorectal Cancer Stratified Medicine Network | International | Non-profit organization | 2018 | 2023 | Non-profit research organization | IIa | <https://www.cancerresearchuk.org/funding-for-researchers/accelerator-award/portfolio-funded-projects-outputs> |
| Actionable targets in clonal progression and systemic spreading of myeloid neoplasms | National | Non-profit organization | 2018 | 2026 | University | IIa | <https://www.airc.it/ricercatori/i-nostri-ricercatori/alessandro-vannucchi> |
| B1MG: Beyond 1 Million Genomes | International | European Commission | 2020 | 2023 | Governmental research organization | Ib | <https://cordis.europa.eu/project/id/951724> |
| BATMAN: Biomolecular Analyses for Tailored Medicine in Acne iNversa | International | European Commission | 2019 | 2022 | Healthcare facility | IIb | <https://www.era-learn.eu/network-information/networks/era-permed/1st-joint-transnational-call-for-proposals-2018/identification-of-markers-for-personal-phenotyping-in-acne-inversa> |
| CAN.HEAL: Building the EU Cancer and Public Health Genomics platform | International | European Commission | 2022 | 2024 | Governmental research organization | Ia | <https://ec.europa.eu/info/funding-tenders/opportunities/portal/screen/how-to-participate/org-details/999999999/project/101080009/program/43332642/details> |
| CancerPrev: Innovative strategies for cancer prevention with focus on sex hormone signaling and chronic inflammation | International | European Commission | 2019 | 2024 | University | Ia | <https://cordis.europa.eu/project/id/859860> |
| Caratterizzazione molecolare del virus pandemico SARS- CoV-2 in Italia | National | National central government | 2020 | 2022 | Governmental research organization | IIIa | [https://www.ccm-network.it](https://www.ccm-network.it/)/progetto.jsp?id=node/2031&idP=740 |
| DECIPHER-ALS: Deconstruct and rebuild phenotypes: a multimodal approach toward personalized medicine in ALS | National | National central government | 2019 | 2023 | University | IIb | <https://prin.mur.gov.it/> |
| DECODE: Defining stratification of patients with C3 Glomerulopathies /Immune complex –mediated glomerular diseases for better diagnosis and tailored treatment | International | European Commission | 2021 | 2024 | Healthcare facility | IIb | <https://www.era-learn.eu/network-information/networks/era-permed/multidisciplinary-research-projects-on-personalised-medicine-2013-pre-clinical-research-big-data-and-ict-implementation-and-user2019s-perspective/defining-stratification-of-patients-with-c3-glomerulopathies-immune-complex-2013mediated-glomerular-diseases-for-better-diagnosis-and-tailored-treatment> |
| Definizione e promozione di programmi per l’implementazione delle azioni centrali di supporto al “Piano per l’innovazione del sistema sanitario basata sulle scienze omiche” | National | National central government | 2019 | 2022 | University | Ic | [https://www.ccm-network.it](https://www.ccm-network.it/)/progetto.jsp?id=node/2047&idP=740 |
| DP3: Diagnosi precoce e prognosi del tumore prostatico, un modello integrato tra biologia e tecnologia | National | Bank group | 2020 | 2023 | Non-profit research organization | IIa | <https://www.fondazionetempia.org/tumore-della-prostata-diventate-protagonisti-della-ricerca> |
| EOSC4Cancer: A European-wide foundation to accelerate Data-driven Cancer Research | International | European Commission | 2022 | 2025 | Governmental research organization | Ib | <https://ec.europa.eu/info/funding-tenders/opportunities/portal/screen/how-to-participate/org-details/984108263/project/101058427/program/43108390/details> |
| Epigenetic modeling/remodeling of cancer metastases and tumor immune contexture to improve efficacy of immunotherapy | National | Non-profit organization | 2018 | 2026 | Healthcare facility | IIa | <https://www.airc.it/ricercatori/i-nostri-ricercatori/michele-maio> |
| Ev-glio: Plasma extracellular vesicles (EVs): the key for precision medicine in Glioblastoma | International | European Commission | 2021 | 2024 | Healthcare facility | IIa | <https://www.era-learn.eu/network-information/networks/era-permed/multidisciplinary-research-projects-on-personalised-medicine-2013-pre-clinical-research-big-data-and-ict-implementation-and-user2019s-perspective/plasma-extracellular-vesicles-evs-the-key-for-precision-medicine-in-glioblastoma> |
| ExACT: The European network staff eXchange for integrAting precision helth in the health Care SysTems | International | European Commission | 2019 | 2024 | University | Ia | [https://cordis.europa.eu/project/id/823995](https://cordis.europa.eu/project/id/826121) |
| Exosomal miRNA signature as prognostic marker in advanced non-small cell lung cancer patients treated with nivolumab | National | National central government | 2018 | 2022 | Healthcare facility | IIa | [https://areapubblica.cbim.it/areapubblica/areaprogetti](https://areapubblica.cbim.it/areapubblica/areaprogetti%20%20(CO-2016-02361470)) |
| Exploiting the gut microbiome for cancer diagnosis and treatment | International | European Commission | 2019 | 2024 | Healthcare facility | IIa | <https://cordis.europa.eu/project/id/825410> |
| Exploring genome/phenome interaction in Multiple Sclerosis | National | National central government | 2018 | 2022 | Healthcare facility | IIb | <https://areapubblica.cbim.it/areapubblica/areaprogetti> |
| FINDINGMS: An integrated approach to predict disease activity in the early phases of Multiple Sclerosis | International | European Commission | 2019 | 2022 | Healthcare facility | IIb | <https://www.era-learn.eu/network-information/networks/era-permed/1st-joint-transnational-call-for-proposals-2018/an-integrated-approach-to-predict-disease-activity-in-the-early-phases-of-multiple-sclerosis> |
| FRAIL-BRAIN: Biological markers of frailty in the physiological and pathological aging brain: correlations with pharmacological frailty | National | National central government | 2018 | 2022 | Healthcare facility | IIb | [https://www.aifa.gov.it/documents/20142/516919/Bando-AIFA-2016-19.07.2018.pdf](https://www.aifa.gov.it/en/archivio-bandi) |
| GDI: Genomic Data Infrastructure | International | European Commission | 2022 | 2026 | Governmental research organization | Ib | <https://ec.europa.eu/info/funding-tenders/opportunities/portal/screen/how-to-participate/org-details/999999999/project/101081813/program/43152860/details> |
| GENOMED4ALL: Genomics and Personalized Medicine for all though Artificial Intelligence in Haematological Diseases | International | European Commission | 2021 | 2024 | University | Ib | <https://cordis.europa.eu/project/id/101017549> |
| GERSOM: Studio di fattibilità per la diagnosi genomica congiunta di rischio genetico e di sensibilità ai nuovi farmaci nelle neoplasie del seno, ovaio e colon | National | National central government | 2019 | 2022 | Governmental research organization | IIa | <https://www.alleanzacontroilcancro.it/progetti/gersom> |
| Health Big Data | National | National central government | 2019 | 2029 | Governmental research organization | Ib | <https://www.alleanzacontroilcancro.it/progetti/health-big-data> |
| HiRisk-HiGain: Rethinking personalized cancer therapy: targeting minimal residual disease in high-risk lymphoma patients | International | European Commission | 2021 | 2024 | Healthcare facility | IIa | <https://www.era-learn.eu/network-information/networks/era-permed/multidisciplinary-research-projects-on-personalised-medicine-2013-pre-clinical-research-big-data-and-ict-implementation-and-user2019s-perspective/rethinking-personalized-cancer-therapy-targeting-minimal-residual-disease-in-high-risk-lymphoma-patients> |
| IC2PerMed: Integrating China in the International Consortium for Personalised Medicine | International | European Commission | 2020 | 2023 | University | Ia | <https://cordis.europa.eu/project/id/874694> |
| ICPerMed: Secretariat for the International Consortium for Personalised Medicine | International | European Commission | 2021 | 2024 | Non-profit research organization | Ia | <https://cordis.europa.eu/project/id/964197> |
| IMAGene: Epigenomic and machine learning models to predict pancreatic cancer: development of a new algorithm to integrate clinical, omics, DNA methylation biomarkers and environmental data for early detection of pancreatic cancer in high-risk individuals | International | European Commission | 2022 | 2024 | Healthcare facility | IIa | <https://www.era-learn.eu/network-information/networks/era-permed/joint-transnational-call-for-proposals-2021-for-201cmultidisciplinary-research-projects-on-personalised-medicine-2013-development-of-clinical-support-tools-for-personalised-medicine-implementation201d/epigenomic-and-machine-learning-models-to-predict-pancreatic-cancer-development-of-a-new-algorithm-to-integrate-clinical-omics-dna-methylation-biomarkers-and-environmental-data-for-early-detection-of-pancreatic-cancer-in-high-risk-individuals> |
| Implementazione nella pratica clinica di un percorso diagnostico integrato basato sulle tecnologie-omiche | National | National central government | 2020 | 2023 | Healthcare facility | IIb | <https://areapubblica.cbim.it/areapubblica/areaprogetti> |
| Integrazione di dati genetici e fenotipici tra il database PROGEMUS e il Registro Italiano SM | National | Non-profit organization | 2022 | 2023 | Healthcare facility | Ib | <https://www.aism.it/sites/default/files/Compendio_Ricerca_2022_0.pdf> |
| INTERVENE: International consortium for integrative genomics prediction | International | European Commission | 2021 | 2025 | University | Ib | <https://cordis.europa.eu/project/id/101016775> |
| iPC: individualizedPaediatricCure: Cloud-based virtual-patient models for precision paediatric oncology | International | European Commission | 2019 | 2023 | For profit research organization | Ib | <https://cordis.europa.eu/project/id/826121> |
| LQTS-NEXT: to the NEXT level of risk prediction in patients with Long QTS Syndrome | International | European Commission | 2020 | 2023 | University | IIb | <https://www.era-learn.eu/network-information/networks/sc1-bhc-04-2018/1st-ejp-rd-joint-transnational-call-for-rare-diseases-research-project-jtc-2019/to-the-next-level-of-risk-prediction-in-patients-with-long-qt-syndrome> |
| LVADSTRAT: Stratification of heart failure patients for cardiac recovery upon cardiac unloading by left ventricular assist device therapy: addressing the molecular, epigenetic, and proteomic changes associated with reverse cardiac remodelling. | International | European Commission | 2019 | 2022 | University | IIb | <https://www.era-learn.eu/network-information/networks/era-permed/1st-joint-transnational-call-for-proposals-2018/stratification-of-heart-failure-patients-for-cardiac-recovery-upon-cardiac-unloading-by-left-ventricular-assist-device-therapy-addressing-the-molecular-epigenetic-and-proteomic-changes-associated-with-reverse-cardiac-remodelling> |
| MEET-AML: Metabolic vulnerabilities for personalized therapeutic approaches in acute myeloid leukemia | International | European Commission | 2020 | 2023 | Healthcare facility | IIa | <https://www.era-learn.eu/network-information/networks/era-permed/personalised-medicine-multidisciplinary-research-towards-implementation/metabolic-vulnerabilities-for-personalized-therapeutic-approaches-in-acute-myeloid-leukemia> |
| Methylation based liquid biopsy to predict molecular residual disease and risk of recurrence in colon cancer patients | National | Non-profit organization | 2019 | 2023 | Healthcare facility | IIa | <https://www.airc.it/ricercatori/i-nostri-ricercatori/federica-di-nicolantonio> |
| MIRACLE: A Machine learning approach to Identify patients with Resected non-small-cell lung cAnCer with high risk of reLapsE | International | European Commission | 2022 | 2025 | Healthcare facility | IIa | <https://www.era-learn.eu/network-information/networks/era-permed/joint-transnational-call-for-proposals-2021-for-201cmultidisciplinary-research-projects-on-personalised-medicine-2013-development-of-clinical-support-tools-for-personalised-medicine-implementation201d/a-machine-learning-approach-to-identify-patients-with-resected-non-small-cell-lung-cancer-with-high-risk-of-relapse> |
| MULTIPLE-MS: Multiple manifestations of genetic and non-genetic factors in Multiple Sclerosis disentangled with a multi-omics approach to accelerate personalised medicine | International | European Commission | 2017 | 2022 | University | IIb | <https://cordis.europa.eu/project/id/733161> |
| NEUDIG: Unveiling the hidden side of NEUrodevelopmental DIsorder Genetics: a multidisciplinary pathway to new molecular diagnoses by integrating genomic, transcriptomic, and functional analyses | National | National central government | 2022 | 2025 | University | IIb | https://prin.mur.gov.it/ |
| Novel predictive biomarkers in the clinical management of ibrutinib-treated chronic lymphocytic leukemia patients: results form an observational prospective clinical trial | National | National central government | 2020 | 2023 | Healthcare facility | IIa | <https://areapubblica.cbim.it/areapubblica/areaprogetti> |
| OncNGS: NGS diagnostics in 21st century oncology: the best, for all, at all times | International | European Commission | 2020 | 2025 | Governmental research organization | Ic | <https://cordis.europa.eu/project/id/874467> |
| Open questions in acute lymphoblastic leukemia | National | National central government | 2019 | 2023 | University | IIa | <https://prin.mur.gov.it/> |
| PARP1 and immune checkpoint inhibition after chemotherapy induction in leiomyosarcoma: a model to unleash immunoresponse | National | Non-profit organization | 2020 | 2024 | Healthcare facility | IIa | <https://www.airc.it/ricercatori/i-nostri-ricercatori/giovanni-grignani> |
| PerMiM: Personalized Mitochondrial Medicine (PerMiM): Optimizing diagnostics and treatment for patients with mitochondrial diseases | International | European Commission | 2020 | 2023 | Healthcare facility | IIb | <https://www.era-learn.eu/network-information/networks/era-permed/personalised-medicine-multidisciplinary-research-towards-implementation/personalized-mitochondrial-medicine-permim-optimizing-diagnostics-and-treatment-for-patients-with-mitochondrial-diseases> |
| PERMIT: PERsonalised MedicIne Trials | International | European Commission | 2020 | 2022 | Governmental research organization | Ia | <https://cordis.europa.eu/project/id/874825> |
| PER-NEPH: Implementation of personalised management in nephrotic syndrome | International | European Commission | 2022 | 2024 | Healthcare facility | IIb | <https://www.era-learn.eu/network-information/networks/era-permed/joint-transnational-call-for-proposals-2021-for-201cmultidisciplinary-research-projects-on-personalised-medicine-2013-development-of-clinical-support-tools-for-personalised-medicine-implementation201d/implementation-of-personalised-management-in-nephrotic-syndrome> |
| PerProGlio: Integrative Personal Omics Profiles in Glioblastoma Recurrence and Therapy Resistance | International | European Commission | 2019 | 2022 | University | IIa | <https://www.era-learn.eu/network-information/networks/era-permed/1st-joint-transnational-call-for-proposals-2018/integrative-personal-omics-profiles-in-glioblastoma-recurrence-and-therapy-resistance> |
| Personalized medicine. Advancing chemical and genomic strategies for relapsed/refractory T-ALL | National | Non-profit organization | 2020 | 2023 | University | IIa | <https://www.gimema.it/fondo-per-le-idee-2019-progetti-finanziati/> |
| PLOT-BD: Personalization of Long-term Treatment in Bipolar Disorder | International | European Commission | 2019 | 2022 | University | IIb | <https://www.era-learn.eu/network-information/networks/era-permed/1st-joint-transnational-call-for-proposals-2018/personalization-of-long-term-treatment-in-bipolar-disorder> |
| PREDICT-Meso: PRE-malignant Drivers Combined with Target-Drug validation in Mesothelioma | International | Non-profit organization | 2019 | 2024 | University | IIa | <https://www.cancerresearchuk.org/funding-for-researchers/accelerator-award/portfolio-funded-projects-outputs> |
| Preserve: AI for new signatures and models for tailored organ preservation approaches in laryngeal and hypopharyngeal cancer | International | European Commission | 2021 | 2024 | Healthcare facility | IIa | <https://www.era-learn.eu/network-information/networks/era-permed/multidisciplinary-research-projects-on-personalised-medicine-2013-pre-clinical-research-big-data-and-ict-implementation-and-user2019s-perspective/ai-for-new-signatures-and-models-for-tailored-organ-preservation-approaches-in-laryngeal-and-hypopharyngeal-cancer> |
| PROPHET: A PeRsOnalized Prevention roadmap for the future HEalThcare | International | European Commission | 2022 | 2026 | University | Ia | <https://cordis.europa.eu/project/id/101057721> |
| PROMPT: Toward PrecisiOn Medicine for the Prediction of Treatment response in major depressive disorder through stratification of combined clinical and -omics signatures | International | European Commission | 2021 | 2024 | University | IIb | <https://www.era-learn.eu/network-information/networks/era-permed/multidisciplinary-research-projects-on-personalised-medicine-2013-pre-clinical-research-big-data-and-ict-implementation-and-user2019s-perspective/toward-precision-medicine-for-the-prediction-of-treatment-response-in-major-depressive-disorder-through-stratification-of-combined-clinical-and-omics-signatures> |
| Pseudomyxoma peritonei (PMP): building a european multicentric cohort to accelerate new therapeutic perspectives | International | Non-profit organization | 2020 | 2025 | Healthcare facility | IIa | <https://www.cancerresearchuk.org/funding-for-researchers/accelerator-award/portfolio-funded-projects-outputs> |
| REGIONS4PERMED: Interregional coordination for a fast and deep uptake of personalised health | International | European Commission | 2018 | 2023 | Non-profit research organization | Ia | <https://cordis.europa.eu/project/id/825812> |
| R-Link: Optimizing response to Li treatment through personalized evaluation of individuals with bipolar I disorder: the R-LiNK initiative | International | European Commission | 2018 | 2023 | Governmental research organization | IIb | <https://cordis.europa.eu/project/id/754907> |
| SINO-EU-PerMed: Widening Sino‐EU policy and research cooperation in Personalised Medicine | International | European Commission | 2020 | 2023 | Non-profit research organization | Ia | [https://cordis.europa.eu/project/id/874556](https://cordis.europa.eu/project/id/874556/it) |
| Strategia Genomica italiana: istituzione di una cabina di regia a supporto dell’iniziativa europea  1+Million Genomes (1+MG) e Beyond 1+MG (B1MG) e del Coordinamento Interistituzionale per la Genomica in Sanità Pubblica | National | National central government | 2021 | 2023 | University | Ib | [https://www.ccm-network.it](https://www.ccm-network.it/) |
| Strategies to overcome acquired resistance to targeted therapies in colorectal cancer | National | Non-profit organization | 2018 | 2022 | Healthcare facility | IIa | <https://www.airc.it/ricercatori/i-nostri-ricercatori/sabrina-arena> |
| SUPERTREAT: Supporting Personalized Treatment Decisions in Head and Neck Cancer through Big Data | International | European Commission | 2020 | 2023 | University | IIa | <https://www.era-learn.eu/network-information/networks/era-permed/personalised-medicine-multidisciplinary-research-towards-implementation/supporting-personalized-treatment-decisions-in-head-and-neck-cancer-through-big-data> |
| SURVEID: Studio pilota per la sorveglianza di potenziali minacce da malattie infettive emergenti (EIDs) di origine virale mediante una piattaforma diagnostica basata sul sequenziamento metagenomico di nuova generazione (mNGS). | National | National central government | 2022 | 2024 | Healthcare facility | IIIa | [https://www.ccm-network.it](https://www.ccm-network.it/) |
| SYMMETRY: Subpopulation heterogeneitY and MicroenvironMEntal engagement as predictors for Treatment Resistance in lYmphoma | International | European Commission | 2022 | 2025 | University | IIa | <https://www.era-learn.eu/network-information/networks/era-permed/joint-transnational-call-for-proposals-2021-for-201cmultidisciplinary-research-projects-on-personalised-medicine-2013-development-of-clinical-support-tools-for-personalised-medicine-implementation201d/subpopulation-heterogeneity-and-microenvironmental-engagement-as-predictors-for-treatment-resistance-in-lymphoma> |
| THRuST: Early detection of relapses in stage III colon cancer patients by longitudinally following a personalized molecular signature from a blood test | International | European Commission | 2018 | 2022 | Non-profit research organization | IIa | <https://www.transcanfp7.eu/index.php/abstract/thrust.html> |
| TOPMESO: A TranslatiOnal Platform for de-orphaning malignant pleural MESOthelioma | International | European Commission | 2019 | 2022 | Healthcare facility | IIa | <https://www.transcanfp7.eu/index.php/abstract/topmeso.html> |

^1^ Thematic categories and sub-categories: I) governance, further divided into Ia) networking and coordination for innovation, Ib) data and infrastructure, Ic) health technology adoption; II) precision medicine, further divided into IIa) cancer, IIb) non-oncological diseases; and III) precision public health, including only one sub-category, namely IIIa) surveillance of infectious diseases.
